# Experience of a resident physician in general surgery basic area on board of an expedition in the amazon river

**DOI:** 10.1590/0100-6991e-20223369-en

**Published:** 2022-11-10

**Authors:** MARINA FANELLI LUCHIARI MILANI, DANIELA SILVESTRE, JULIANA MARIM

**Affiliations:** 1 - Hospital Universitario São Francisco de Assis, Residência Médica - Bragança Paulista - SP - Brasil; 2 - Hospital Universitario São Francisco de Assis, Cirurgia Pediátrica - Bragança Paulista - SP - Brasil; 3 - Hospital Universitario São Francisco de Assis, Psiquiatria - Bragança Paulista - SP - Brasil

**Keywords:** Education, Medical, Internship and Residency, Rivers, Educação Médica, Internato e Residência, Características de Residência, Navios

## Abstract

Located in areas of difficult access, the riverside population of the upper Amazon River has a great demand for health care, whether in the scope of basic health promotion or in general or specialized medical care, surgical procedures, dental and pharmaceutical care. Taking this to consideration, the Barco Hospital Papa Francisco project was conceived and implemented, which aims to provide health care to riverside communities through expeditions that safely transfer health resources to populations located on the banks of the river. Having participated in one of the expeditions, it was possible to carry out a survey of data regarding the attendance and writing of a personal report on the impact on the professional activity. The expedition allowed the performance of a large number of surgical procedures in a condensed period, covering a wide variety of technical approaches essential to the performance of the general surgeon, among them, we can mention inguinal and incisional hernioplasties, umbilical and inguinal herniorrhaphy, postectomy and tubal ligation, lipoma excision, sebaceous cyst excision, nevi excision, among others (data available in the vessels Wareline^®^ system).

## RESUMO

Localizadas em áreas de difícil acesso, a população ribeirinha do alto rio Amazonas apresenta grande demanda assistencial de saúde, seja ela no âmbito de promoção de saúde básica seja no atendimento médico geral ou especializado, procedimentos cirúrgicos, atendimentos odontológicos e farmacêutico. Frente a isso, foi idealizado e concretizado o projeto do Barco Hospital Papa Francisco, que visa o atendimento de saúde às comunidades ribeirinhas através de expedições que deslocam os recursos de saúde de forma segura até as populações situadas às margens do rio. Tendo participado de uma das expedições, foi possível realizar levantamento de dados referente aos atendimentos e redação de relato pessoal sobre o impacto na atividade profissional. A expedição proporcionou a realização de grande número de procedimentos cirúrgicos em um período condensado, abrangendo ampla variedade de abordagens técnicas imprescindíveis à atuação do cirurgião geral, entre elas, pode-se citar hernioplastias inguinal e incisional, herniorrafia umbilical e inguinal, postectomia e laqueadura, exérese de lipomas, exérese de cisto sebáceo, exérese de nevos, entre outras (dados disponíveis em sistema Wareline^®^ da embarcação).

## METHOD

This is a personal report and collection of specific data from a medical expedition carried out at a Hospital Boat on the Amazon River, Pará, Brazil, between 12/10/2019 and 12/17/2019 (6^th^ Expedition). Official data were provided by the Associação Lar São Francisco (ALSF) through registration by the Wareline^®^ hospital system and analysis of articles published in research on the Google Academic platform.

## DISCUSSION

### Overview

In view of the health demand of the riverside population of the upper Amazon River and the dangers of navigating small boats over long distances in the waters of Northern Brazil, there was a need to provide an adequate place for patients to access the health system, surgical procedures, adequate general or specialized medical, dental, and pharmaceutical care, through the provision of resources to communities throughout the river^1^ ^,^ ^2^.

In the literature, there are few reports of boats equipped only for medical care and few naval vessels are able to perform surgical procedures on their premises. Hospital ships of the American Navy (Comfort), the Brazilian Navy (Hospital Assistance Ships) have been described, among few others that have such capacity because they contain operating rooms or surgical centers in their facilities¹.

Thus, the project for the Barco Hospital Papa Francisco (Pope Francis Hospital Boat) was born, which joined the few vessels in the world to house surgical procedures and to meet the medical demands of the population of the upper Amazon River, being the only one to do so in this locality³.

Through the participation of the Pope Francis Hospital Boat project, it was possible to write a personal report about the experiences acquired during the expedition period.

### Pope Francis Hospital Boat

Conceived by the São Francisco de Assis Association and Fraternity in the Providence of God, the Pope Francis Hospital Boat is based in the city of Óbidos, state of Pará, and operates in the Pará Amazon Basin (upper Amazon River). It aims to serve 700,000 people from riverside communities in the municipalities of Alenquer, Alemrim, Belterra, Curruá, Faro, Juruti, Monte Alegre, Óbidos, Oriximiná, Prainha, Santarém, Terra Santa, and others.

The construction of the Hospital Ship was conceived during the World Youth Day 2013, in Rio de Janeiro-RJ, in a conversation between Pope Francis and the director of Associação Lar São Francisco. With the aim of promoting care for the riverside population, the project works with the provision of health resources to people in an attempt to avoid accidents along the way to which they are subjected in the waters of the great rivers of the north and, thus, provide a minimum access to health for the most distant populations.

The project came to life with resources arising from a class action agreement approved in 2013 by the Regional Labor Court of Campinas-SP, between the companies Raízen Combustíveis S/A (formerly Shell Química) and Basf S/A and the Labor District Attorney Office.

With approximately 35 meters in length, the vessel has three medical offices, one dental office, one operating room, one ophthalmological room, one analysis laboratory, one medication room, one vaccination room, three infirmary beds, and exam equipment (X-ray, ultrasound, echocardiogram, mammography, treadmill, and electrocardiogram). The team for each expedition is composed of 10 permanent crew members (commander, cook, on-board assistants, pharmacist, vessel manager, among others) and, generally, 20 health care volunteers whose activities include basic health care (dental care and different medical areas), prevention and early diagnosis of some types of cancer, and surgical procedures of low and medium complexity.

To complement the assistance provided to the communities, there are also two “ambulanchas” (smaller boats equipped as ambulances), one of them responsible for triage work to optimize the assistance provided on the hospital boat, and the other equipped with urgency and emergency equipment and which acts as a backup for any serious complications encountered during the consultations (data from 2019).

### Expeditions

Expeditions occur on a monthly basis. In 2019, eight were carried out (since the opening of Hospital Boat, in February), with a total of 23,950 consultations divided between all the areas offered by the boat (clinical, surgical, dental, ophthalmological consultations; imaging tests; laboratory tests; surgical procedures; drug delivery) (Table 1).

**Table 1:** Total consultations at the Hospital Boat in 2019.

Clinical / Surgical Consultations	6.093
Ophthalmological Consultations	1.371
Dental Appointments / Procedures	1.080
X ray	1.038
Mammography	349
Ultrasound	1.840
Clinical Analysis (laboratory tests)	4.443
Ophthalmology	1.972
Electrocardiogram	487
Clinical / Surgical Admissions	177
Minor Surgeries	251
Medium Complexity Surgeries	158
Childbirths / C-sections	1
Delivery of Medicines	4.690

### Perioperative care

The selection of patients eligible for a surgical procedure begins in the form of triage before they enter the boat’s facilities. The screening is carried out by the vessel’s permanent crew, who assess the demands of the patients and, if they are surgical, forward them to the surgical team for evaluation. Patients are indicated for procedures on immediate demand (performed on the same day) if they are in good general condition, without decompensated comorbidities, and fasting from water and food. If they do not fulfill these criteria, they are instructed to return on another day of service to the boat to undergo the procedure; if the demand cannot be approached, the procedure on the vessel is contraindicated.

After evaluation and surgical indication, if the procedure corresponds to a medium-sized or pediatric age group, an anesthetic evaluation takes place for safer submission of the patient to spinal anesthesia and sedation. In these cases (medium procedure), patients sign anesthetic and surgical consent forms.

For the surgical procedure, the following are available: sterile drapes and disposable gowns, surgical boxes chemically sterilized on the boat’s facilities (Sterile Materials Center), electric scalpel, absorbable and non-absorbable threads, surgical meshes for hernia repair, among other equipment.

The immediate postoperative period for patients undergoing spinal anesthesia and sedation is performed in beds intended for post anesthetic recovery, in the form of surgical hospitalization. Upon improvement of the anesthetic effects, the patient is discharged from the boat, with return to a health unit in Óbidos-PA for postoperative follow-up and removal of stitches.

During the consultations, patients who presented complications in the intra or postoperative period, whether surgical or related to anesthesia, were referred to the Santa Casa de Misericórdia in the city of Óbidos-PA for follow-up with the local attending physician, in daily visits and under conducts guided by the surgical team.

Among the patients submitted to medium-sized procedures, some were already on the surgical waiting list of the Public Unified Health System (SUS) to undergo the intervention in Santarém-PA, and part of them were referred by the Regional Health Department to be treated on the Boat due to the long delay in the SUS queue.

The other procedures (minor surgeries in adults), which corresponded to the vast majority of the population’s demand and, consequently, of the interventions, were performed with local anesthesia, without the need for pre-anesthetic evaluation or fasting.

### Personal Report

The period of work at the Hospital Boat provided a large number of surgical procedures that were performed by the surgical team composed of a first-year resident of General Surgery Basic Area and by a certified general surgeon.

The expedition provided a significant number of surgical procedures in a condensed period, covering a wide variety of technical approaches essential to the training of the general surgeon. Among them, we can mention inguinal hernia repair (Lichtenstein, Shouldice, Bassini, McVay techniques), umbilical herniorrhaphy (Mayo technique), and incisional hernias, postectomy and tubal ligation, lipoma excision, sebaceous cyst excision, excision of nevi, among others (data available in the vessel’s Wareline® system) (Tables 2 and 3).

**Table 2:** Surgical data from the 6 ^th^ expedition.

Surgical size of procedures	Number
Minor Surgeries	86
Medium Complexity Surgeries	30
Total Procedures	116

**Table 3:** Surgical procedures performed on the 6 ^th^ expedition.

Medium complexity procedures	Number of admissions
Abscess drainage	2
Excision of skin and adnexal tumor / sebaceous cyst	1
Frenectomy	1
Fulguration/chemical cauterization of skin lesions	1
Tubal ligation	6
Postectomy	3
Repairing other hernias	13
Resection of synechiae	1
Removal of foreign body from the auditory cavity	1
Total	30

The expressive number of procedures made it possible to deepen the operative techniques and development of surgical skills during the eight days of care. In addition to medium-sized surgeries, the performance of small procedures aided in the consolidation of skills.

Regarding the demand of the population served in the upper Amazon, most patients showed a need for aesthetic procedures (removal of nevi, lipomas, and cysts on the face, back, and limbs), which are considered low-complexity procedures. There was also a high incidence of inguinal, umbilical, and incisional hernias and significant demand for tubal ligation, classified as medium complexity procedures.

On a personal level, experiencing a reality so different from the country side of the state of São Paulo and having come from a private university, where health resources with assistance at all levels of complexity are available at all times, had a great and reflective impact about privileges. On the other hand, the natural and cultural wealth experienced and the possibility of rich contact with nature after arduous moments on duty demonstrated the clash with the lifestyle of metropolitan regions.

In addition to technical and personal development, it was possible to observe some points that need improvement so that there is better postoperative recovery and better follow-up of patients undergoing procedures.

### Obstacles

Some of the difficulties faced and improvement points observed during the eight days of care include:

- Need of sutures removal after the procedure;
- Inability to forward material for anatomopathological examination; and
- Loss of patient follow-up.

Despite providing care to the population by traveling to communities, postoperative patients still had the need to seek medical assistance or health professionals trained to have sutures removed and possible surgical complications evaluated.

In addition, considering the possibility that some of the lesions analyzed by physical examination and anamnesis that underwent excision showed signs of malignancy, there was a limitation of the system in the analysis of excised specimens and difficulty for patients with probable neoplasia (mostly skin) to continue treatment. This is due to the fact that the anatomopathological analysis (and consequently of the diagnosis) is carried out by sending the specimens with the patients themselves to health institutions that are located in areas of difficult access, which contradicts the purpose of the entire project. There is also difficulty in following up neoplastic involvement in case the sample returns positive for malignancy.

Most patients did not receive adequate postoperative follow-up in the form of daily medical visits to identify early complications. Also, the surgical team does not receive late postoperative information from patients undergoing their procedures, which is important for surgeons.

These items were reported to the sponsoring association with a view to improving this system and reducing the mobilization of postoperative patients, in addition to improving late postoperative follow-up and providing appropriate follow-up for possible cancer patients.

## CONCLUSION

The Pope Francis Hospital Boat project mostly manages to fulfill its mission: to promote health to populations that do not have adequate access to everyday resources and offer specialized care, such as the specialty of general surgery. As a bonus to the professional work carried out, expeditions allow the technical and personal development and growth of its volunteers. Although not the main focus, this bonus becomes extremely attractive and satisfying.

Despite the points to be improved so that the support offered by the vessel is adequate, the project offers easy and safe care in the promotion of health for the riverside populations of the upper Amazon River and complements its scope by improving professional and personal life of its crew through the rich experience provided to the first-year resident of General Surgery Basic Area during her time on board.
